# Study of vision‐related resting‐state activity in suprasellar tumor patients with postoperative visual damage

**DOI:** 10.1002/brb3.3462

**Published:** 2024-03-11

**Authors:** Fuyu Wang, Tao Zhou, Peng Wang, Yanyang Zhang, Jinli Jiang

**Affiliations:** ^1^ Department of Neurosurgery The First Medical Center, Chinese PLA General Hospital Beijing China; ^2^ Department of Neurosurgery Hainan Hospital of Chinese PLA General Hospital Sanya China

**Keywords:** functional connectivity, resting‐state functional magnetic resonance imaging, suprasellar tumor, visual damage

## Abstract

**Introduction:**

The objective of this study was to investigate changes in vision‐related resting‐state activity in patients with suprasellar tumors (ST) who experienced vision deterioration after surgery.

**Methods:**

Twelve patients with ST and vision deterioration after surgery were included in the study. Resting‐state functional connectivity (FC) was compared before and after surgery using a seed‐based analysis with a priori specified regions of interest (ROIs) within the visual areas. The differences between the two groups were identified using a paired *t*‐test.

**Results:**

The data showed a decrease in FC within and between the dorsal and ventral pathways, as well as in the third pathway in ST patients. The middle temporal visual cortex (MT+) showed a decreased FC with more regions than other visual ROIs. The data also revealed an increase in FC between the visual ROIs and higher‐order cortex. The superior frontal gyrus/BA8 showed an increased FC with more ROIs than other high‐order regions, and the hOC4d was involved in an increased FC with more high‐order regions than other ROIs.

**Conclusions:**

The study results indicate significant neural reorganization in the vision‐related cortex of ST patients with postoperative vision damage. Most subareas within the visual cortex showed remarkable neural dysfunction, and some highe‐order cortex may be primarily involved in top‐down control of the subareas within the visual cortex. The hot zones may arise in the processing of “top‐down” influence.

## Graphical abstract

The results show significant neural reorganization in the vision‐related cortex of ST patients with postoperative vision damage. Most subareas within the visual cortex exhibited remarkable neural dysfunction. Some higher‐order cortex may be involved in top‐down control of the subareas within the visual cortex. The hot zones may arise in the processing of “Top‐down.”

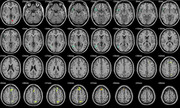

## 1 INTRODUCTION

Neurofunctional reorganizations can occur after visual deprivation during developmental periods. Congenitally blind (CB) individuals exhibit decreased functional connectivity (FC) between the visual cortex and high‐order areas (Yu et al., 2008). Early blindness (EB) results in increased FC between the visual cortex and the inferior frontal triangular gyrus (Liu et al., 2007). In comparison with CB, late blindness (LB) exhibits different FC patterns within the occipital cortex (Büchel et al., 1998). Wen et al. (2018) and Collignon et al. (2013) demonstrated that LB can present with impaired vision‐motor function and top‐down influences. Qin et al. (2015) revealed that EB exhibits more neurofunctional reorganization than LB.

Compared with LB, patients with late partially damaged vision (LPDV) show significant neural reorganization within and between the vision cortex and higher‐order areas. Some studies were conducted in prechiasmal lesions. Bola et al. (2014) showed that the blindness with partial optic nerve damage is caused not only by primary tissue injury but also by a disruption of brain networks. Sanda et al. (2018) showed decreased cortical thickness in the dorsal area V3d in patients with central and peripheral vision loss. Wu et al. (2015) showed decreased FC within the visual cortex and abnormal correlations between the visual cortex and nonvisual systems in acute optic neuritis. The suprasellar mass lesion might damage the optic nerve, chiasma, and optic tract; we called this around‐chiasmal lesions. Qian et al. (2016) showed remarkable neural dysfunction in most subareas within the visual cortex and enhanced FC between some visual cortex and the thalamic pulvinar in pituitary adenoma (PA) patients with visual damage. They proposed that there is a connection between the visual cortex and higher‐order cognitive areas (Qian et al., 2016). Some authors investigated FC in patients with postchiasmal lesions. Guo et al. (2014) revealed that the hemianopia patients with mild occipital stroke presented enhanced connectivity owing to new connections. Pedersini et al. (2020) demonstrated that a functional reorganization of the brain in hemianopic patients occurred, compensating for the general reduced connectivity. Gallina et al. (2022) showed that postchiasmal lesions in posterior cortices specifically lead to stronger impairments of FC in the alpha range. Allaman et al. (2021) revealed that spontaneous α‐band interactions of visual areas are associated with the severity of visual field deficits in patients with postchiasmal lesion outside of the occipital lobe. This may target effective therapeutic interventions for hemianopic patients.

The two‐visual‐pathway theory is a widely accepted model of visual information processing. The ventral pathway projects along the ventral brain surface, where form/object vision is processed. The dorsal pathway projects along the dorsal brain surface, where spatial/motion vision is processed. Recently, a third visual pathway has been suggested, which projects from the early visual cortex, via motion‐selective areas, into the superior temporal sulcus. The third visual pathway is assumed to function in social perception (Pitcher & Ungerleider, 2021).

“Top‐down” refers to cognitive influences and higher‐order representations that have an effect on earlier brain areas when processing information. The bottom‐up flow of visual signals originates from V1 and ascends along two pathways before arriving at higher‐order areas. Higher‐order areas exert a reciprocal top‐down modulation for every bottom‐up information (Gilbert & Li, 2013; Xiong et al., 2016).

Resting‐state functional magnetic resonance imaging (RS‐fMRI) based on BOLD signals has been successfully used to explore the neural function of the human brain (Auer, 2008; Jiang et al., 2016). FC is an effective method for studying the neurofunctional interaction between distant brain regions (Friston et al., 1993). FC based on a region of interest (ROI) is an efficient tool to focus on a prior brain region before researchers calculate the temporal correlation between neurophysiological events in remote regions (Fox & Raichle, 2007).

In the clinic, suprasellar tumors (ST) have a close relationship with the visual apparatus. Some patients may suffer acute vision damage after surgery, which may be related to the manipulation of the optic nerve or injury of the feeding artery during the surgery. As mentioned above, there are different neurofunctional reorganization patterns between CB or EB, LB, and LPDV. However, the changes in visual‐related neurofunction after postoperative acute vision damage (AVD) have not yet been explored. Therefore, we recruited 12 ST patients with AVD 1 week after the operation. We analyzed RS‐fMRI data using a priori defined ROIs located within the occipital cortex, including V1, V2, hOC3d, hOC3v, hOC4d, hOC4v, the middle temporal visual cortex (MT+), and the fusiform gyrus (FG). V1 and V2 are considered to be early visual cortex. hOC3d, hOC4d, and MT+ are within the dorsal visual stream areas. hOC3v, hOC4v, and FG are within ventral visual stream areas. The MT+ is an area specialized for the procession of motion vision (Tootell et al., 1995; de Jong et al., 1994). FG is an area with strong preference for faces perception, object recognition, and reading (Weiner & Zilles, 2016). The aim of our study was to investigate changes in vision‐related resting‐state activity among patients with ST who developed postoperative AVD. Additionally, we aim to explore the neural plasticity within and between the visual cortex and higher cognitive networks following AVD.

## 2 MATERIALS AND METHODS

### 2.1 Subjects

Twelve patients with ST who had suffered from AVD after transcranial tumor resection (lateral subfrontal approach) were enrolled in this study. The inclusion criteria were: age between 18 and 65 years; absence of ophthalmologic diseases; absence of intracranial lesions involving the visual pathway or cortex; a vision acuity examination performed 1 week after the operation that showed decreased vision acuity by at least 0.2 unilaterally; and no postoperative complications such as intracranial hematoma, hydrocephalus, high fever, hypopituitarism, or severe electrolyte imbalance. This study was approved by the Ethics Committee of the Chinese PLA general hospital, and all patients provided written informed consent.

### 2.2 Data acquisition

MRI was performed 1 day before and 1 week after the operation on a 1.5 T MR system (Espree, Siemens Medical Solution, Erlangen, Germany) in the diagnostic room of the iMRI brain suite (Chen et al., 2012). To limit head movement, a foam pad was used, and earplugs were provided to decrease noise during the scan. During the RS‐fMRI scan, patients were instructed to remain still, keep their eyes closed, and not think about anything. RS‐fMRI data were acquired using an echo‐planar image pulse sequence with the following parameters: 26 axial slices, slice thickness of 4.5 mm, flip angle of 90°, field of view (FOV) of 224 × 224 mm, voxel size of 3 mm, repetition time (TR) of 2000 ms, and echo time (TE) of 45 ms. A T1‐weighted sagittal anatomical image was also acquired using a gradient echo sequence with the following parameters: 192 slices, slice thickness of 1 mm, inversion time of 1100 ms, flip angle of 15°, number of excitations of 1, FOV of 256 × 256 mm, voxel size of 1 mm, TR of 1970 ms, and TE of 2.39 ms.

### 2.3 Clinical and neuroophthalmologic assessments

We evaluated the cognitive function of all subjects using the mini‐mental state examination before the surgery. We also performed neuroophthalmologic examinations on the patients within two days before the surgery and 1 week after the surgery. The examinations included testing the best‐corrected visual acuity for distance using the E chart and reporting the results in the decimal scale. We also performed ophthalmic fundus examinations using a nonmydriatic retinal camera from Topcon, Japan.

### 2.4 RS‐fMRI analysis

#### 2.4.1 Data preprocessing

We preprocessed the RS‐fMRI data using SPM8 (http://www.fil.ion.ucl.ac.uk/spm) and the DPARSF pipeline analysis toolbox (http://www.restfmri.net/) (Yan & Zang, 2010). We discarded the first ten volumes to allow for signal equilibrium, and then applied slice timing correction, head motion correction, normalization, smoothing, linear trend removal, and filtering (0.01−0.08 Hz) to the data.

#### 2.4.2 Analysis of FC

We selected 16 ROIs from the literature (Amunts et al., 2000; Caspers et al., 2013; Kolster et al., 2010; Kujovic et al., 2013; Rottschy et al., 2007), which were defined as 6‐mm radius spheres in both hemispheres (Table 1). All 16 seeds were chosen within the occipital cortex (V1, V2, hOC3d, hOC3v, hOC4d, hOC4v, MT+, and FG).

**TABLE 1:** Regions of interest (ROIs).

	Left			Right			Literature reference
	*X*	*Y*	*Z*	*X*	*Y*	*Z*	
Regions							
V1	–10	–77	3	20	–73	2	Amunts et al. (2000)
V2	–13	–75	6	23	–71	6	Amunts et al. (2000)
hOC3d	–15	–97	23	17	–95	24	Kujovic et al. (2013)
hOC3v	–20	–88	–3	26	–84	–4	Rottschy et al. (2007)
hOC4d	–17	–95	29	19	–94	29	Kujovic et al. (2013)
hOC4v	–29	–84	–7	34	–80	–8	Rottschy et al. (2007)
MT	–48	–75	8	46	–78	6	Kolster et al. (2010)
FG	–30	–76	–9	33	–73	11	Caspers et al. (2010)

Before computing functional connectivity, we removed nonneuronal‐related covariates (i.e., the six parameters of head motion correction, the average time courses of the whole brain [global mean signal], the average time courses within the white matter mask, and the average time courses within the cerebral spinal fluid [CSF] mask) from the preprocessed data using linear regression analysis. We then smoothed the images using a 6‐mm full width at half maximum (FWHM) Gaussian kernel. The FC was calculated between each seed region and each voxel within the whole‐brain mask. We transformed the resulting individual FC maps into z‐maps using Fisher's z‐transformation to improve data normality. We used a voxel‐wise paired *t*‐test to determine the brain regions that presented significant differences in correlation between the pre‐ and postoperation groups for each seed region. We corrected for multiple comparisons using the AlphaSim method in REST, with a corrected threshold of *p* < .05 (uncorrected threshold of *p* < .001 and a minimum of 40 voxels in a cluster).

## 3 RESULTS

### 3.1 Studied population

Twelve patients (4 males and 8 females) with a mean age of 40.9 years (range: 19−53 years) were recruited based on the inclusion criteria. Table 2 summarizes the main demographic and clinical characteristics of the patients.

**TABLE 2:** The main demographic and clinical characteristics of the patients.

No.	Age(years)	diagnosis	Operation side	Visual acuity(preop/postop)	
				L	R
1	44	TSM	R	1.0/1.0	0.7/0.5
2	53	TSM	R	0.8/0.8	0.4/0.1
3	50	TSM	L	0.8/0.5	1.0/1.0
4	19	CP	L	0.4/0.1	0.8/0.8
5	49	CP	L	0.4/0.1	0.5/0.4
6	43	PA	L	0.8/0.6	1.0/1.0
7	23	CP	L	0.8/0.6	0.6/0.6
8	36	CP	R	1.0/1.0	0.8/0.5
9	41	CP	R	1.0/0.8	1.0/0.5
10	37	CP	R	0.7/0.6	0.5/0.1
11	46	PA	L	0.3/0.1	0.4/0.3
12	50	CP	R	0.7/0.7	0.6/0.1

PA: pituitary adenoma; CP: craniopharyngioma; TSM: tuberculum sellar meningioma; R: right; L: left.

### 3.2 Ophthalmologic evaluation

Table 2 presents the results of the ophthalmologic evaluation.

### 3.3 RS‐fMRI analysis

#### 3.3.1 Decreased FC in the patients

**FIGURE 1:**
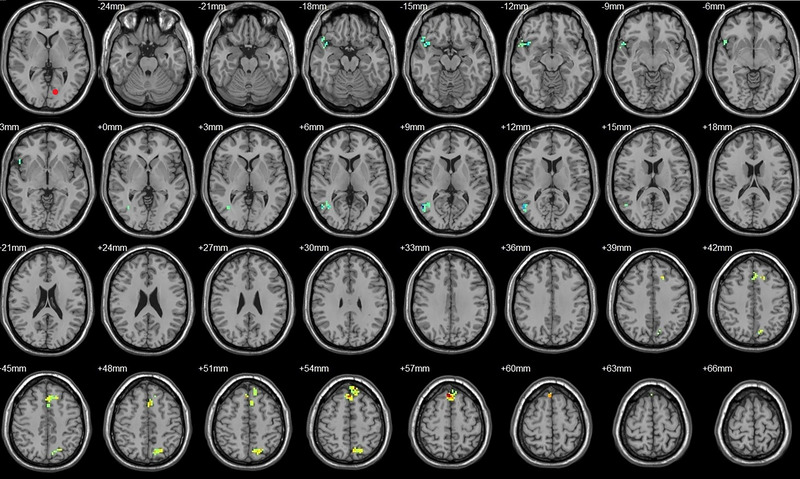
Brain regions showed significantly different FCs with the left V1 in STs (postop vs. preop). The red circle in the left upper corner indicates the seed region.

**FIGURE 2:**
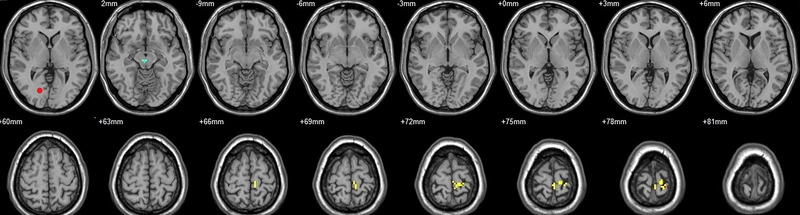
Brain regions showed significantly different FCs with the right V1 in STs (postop vs. preop). The red circle in the left upper corner indicates the seed region.

**FIGURE 3:**
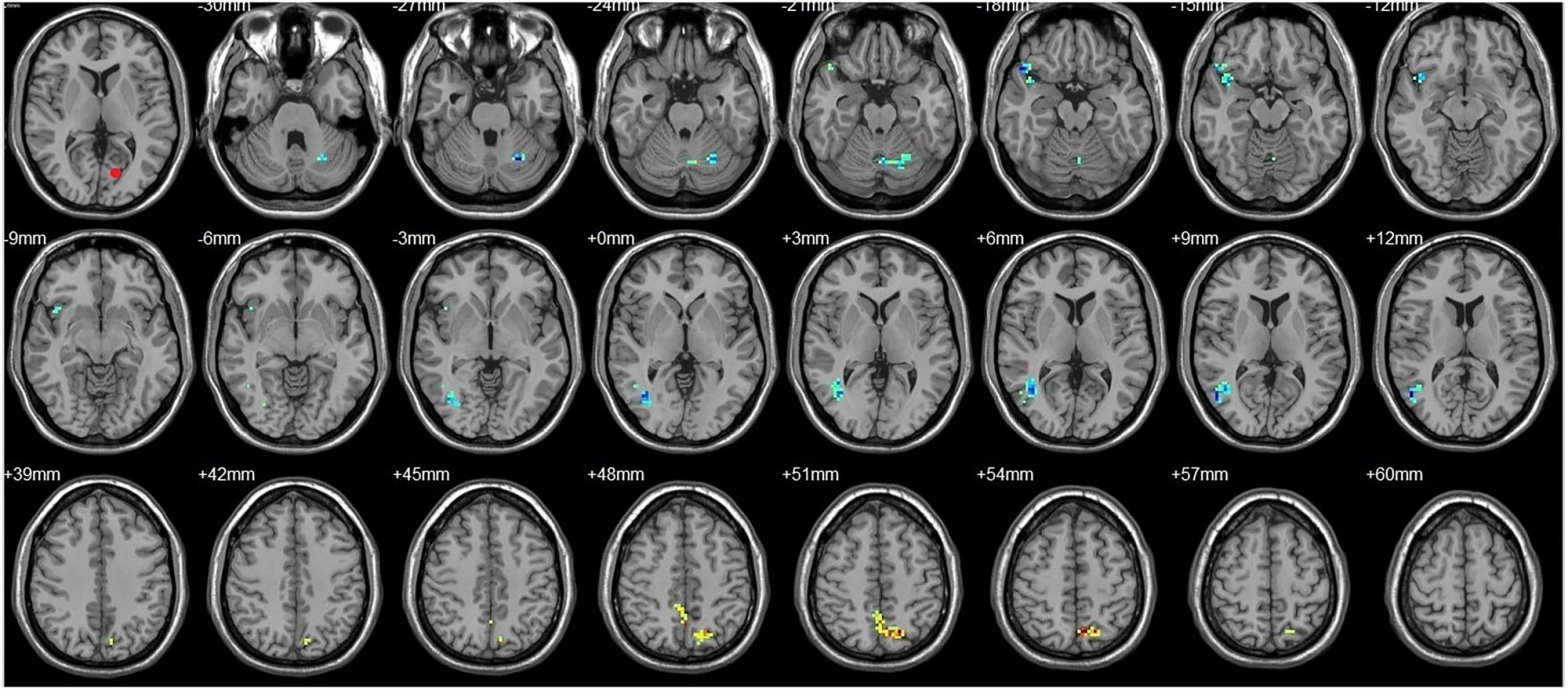
Brain regions showed significantly different FCs with the left V2 in STs (postop vs. preop). The red circle in the left upper corner indicates the seed region.

Compared to their preoperative state, the patients exhibited decreased functional connectivity (FC) with various brain regions after surgery. Specifically, decreased FC with left V1 was observed in the right superior temporal gyrus/BA 38 and right middle temporal gyrus (Figure 1, Table 3),with right V1 in the midbrain (Figure 2, Table 3), with left V2 in the declive and right Brodmann area 19 (Figure 3, Table 3), with left hOC3d in the right thalamus (Figure 5, Table 3), with right hOC3d in the left Brodmann area 18 (Figure 6, Table 3), with right hOC3v in the right Brodmann area 41 (Figure 8, Table 3), with left hOC4d in the right posterior cingulate (Figure 9, Table 3), with right hOC4d in the left lingual (Figure 10, Table 3), with left hOC4v in the left inferior parietal lobule (L) (Figure 11, Table 3), with left MT+ in the left superior temporal gyrus, right middle occipital gyrus, and postcentral gyrus/BA 3 (Figure 12, Table 3), with right MT+ in the left lingual gyrusl, right fusiform, right lingual gyrus, and precuneus/BA 7 (Figure 13, Table 3), and with left FG in the right lingual (Figure 14, Table 3). All these figures were edited to save the space and better illustrate the results, and the original figures could be found in the supplemental files.

**TABLE 3:** Decreased FC after operation.

Seed	Brain region	Peak intensity	Peak MNI coordinate	Cluster size (voxels)
V1 (L)	Superior temporal gyrus/BA 38 (R)	–9.1388	45 12 −15	47
	Middle temporal gyrus (R)	–12.6181	51 −63 9	43
V1 (R)	Midbrain	–7.2873	6 −21 −21	47
V2 (L)	Declive	–9.0791	–27 −63 −27	42
	Brodmann area 19 (R)	–8.5955	51 −66 12	89
hOC3d (L)	Thalamus (R)	–9.099	21 −27 9	45
hOC3d (R)	Brodmann area 18 (L)	–7.7773	–3 −90 21	55
hOC3v (R)	Brodmann area 41 (R)	–7.6542	45 −30 12	43
hOC4d (L)	Posterior cingulate (R)	–8.4899	18 −60 18	41
hOC4d (R)	Lingual (L)	–7.3294	–21 −60 −6	57
hOC4v (L)	Inferior parietal lobule (L)	–7.4352	–57 −30 42	82
MT (L)	Superior temporal gyrus (L)	–6.7223	–54 −27 15	97
	Middle occipital gyrus (R)	–9.7355	33 −72 33	69
	Postcentral gyrus/BA 3	–6.1281	–36 −33 57	43
MT (R)	Lingual gyrus (L)	–10.2504	–6 −96 −6	41
	Fusiform (R)	–7.1196	18 −36 −18	41
	Lingual gyrus (R)	–8.0886	24 −84 −9	54
	Precuneus/BA 7	–6.7487	–18 −84 36	46
FG (L)	Lingual (R)	–7.5297	12 −54 3	62

#### 3.3.2 Increased FC in the patients

**FIGURE 4:**
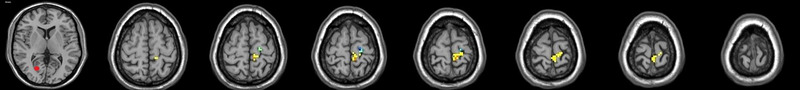
Brain regions showed significantly different FCs with the right V2 in STs (postop vs. preop). The red circle in the left upper corner indicates the seed region.

**FIGURE 5:**
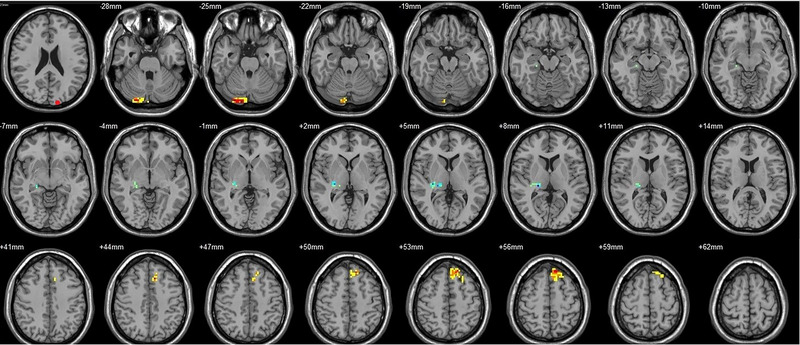
Brain regions showed significantly different FCs with the left hOC3d in STs (postop vs. preop). The red circle in the left upper corner indicates the seed region.

**FIGURE 6:**
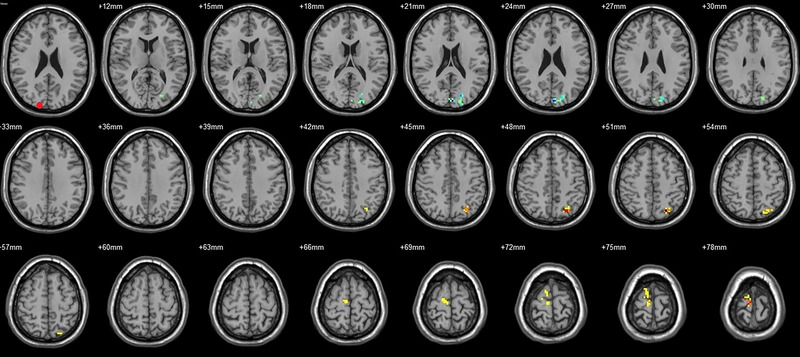
Brain regions showed significantly different FCs with the right hOC3d in STs (postop vs. preop). The red circle in the left upper corner indicates the seed region.

**FIGURE 7:**
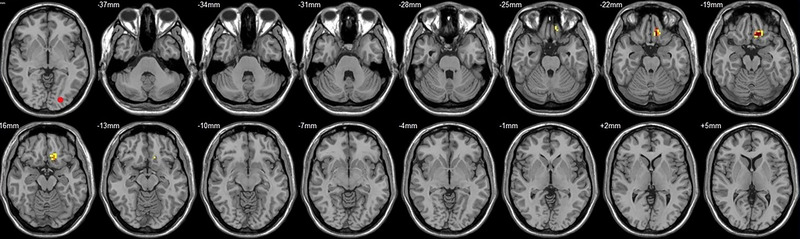
Brain regions showed significantly different FCs with the left hOC3v in STs (postop vs. preop). The red circle in the left upper corner indicates the seed region.

**FIGURE 8:**
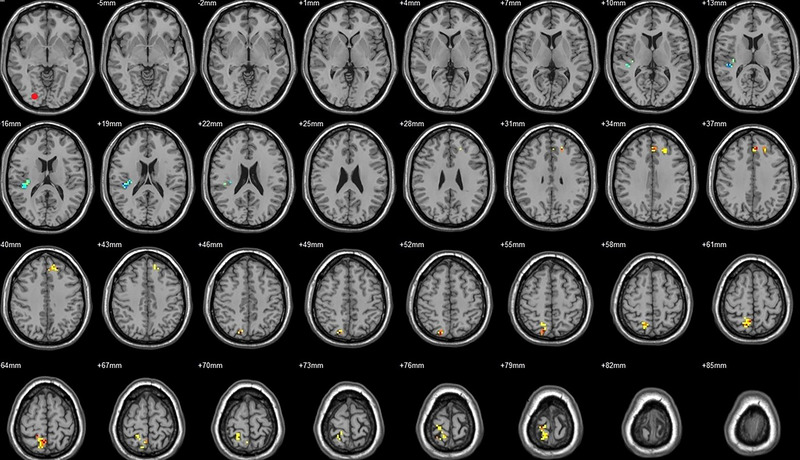
Brain regions showed significantly different FCs with the right hOC3v in STs (postop vs. preop). The red circle in the left upper corner indicates the seed region.

**FIGURE 9:**
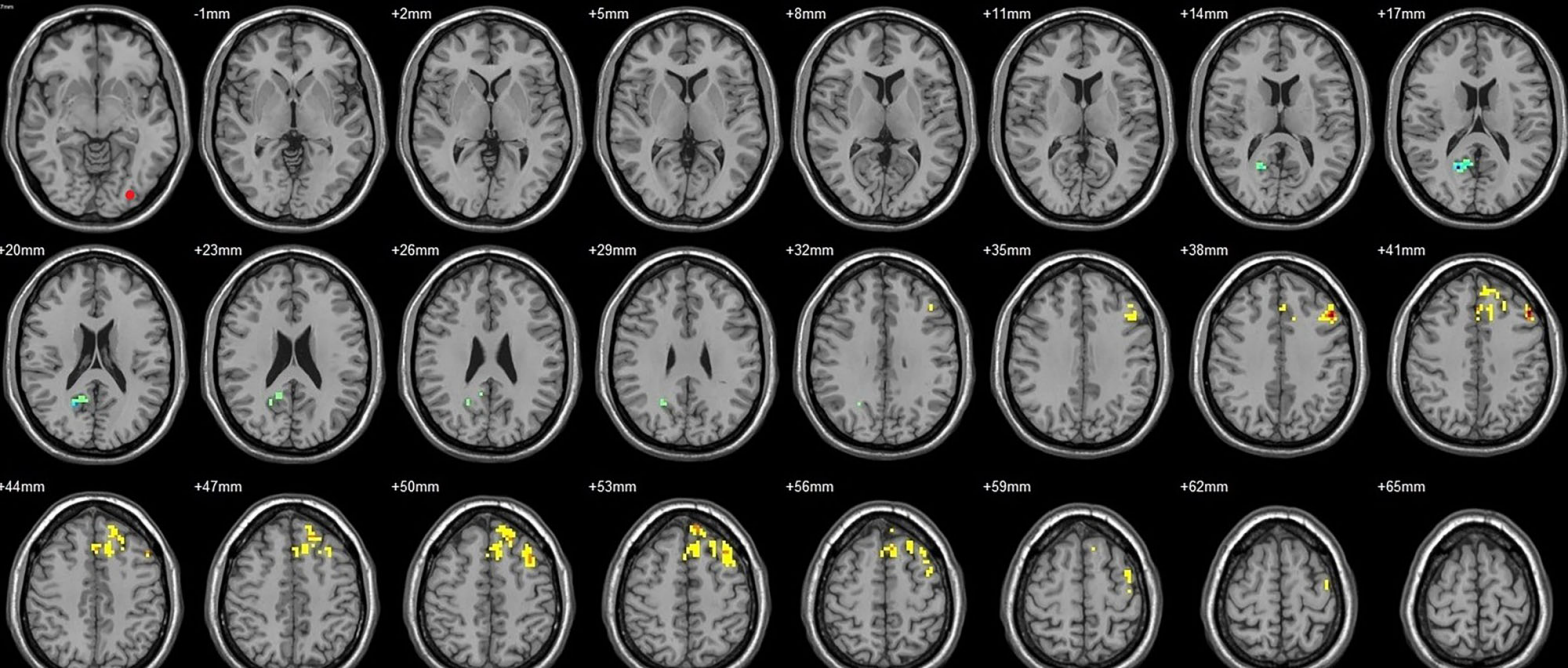
Brain regions showed significantly different FCs with the left hOC4d in STs (postop vs. preop). The red circle in the left upper corner indicates the seed region.

**FIGURE 10:**
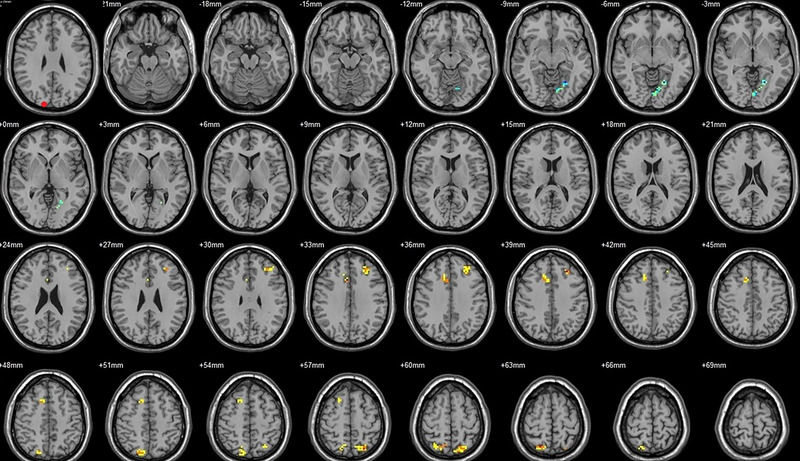
Brain regions showed significantly different FCs with the right hOC4d in STs (postop vs. preop). The red circle in the left upper corner indicates the seed region.

Compared to their preoperative state, the patients exhibited increased functional connectivity (FC) with various brain regions after surgery. Specifically, with the left V1, increased FC was observed in the left superior parietal lobule/BA 7 and right superior frontal gyrus/BA 8 (Figure 1, Table 4); with the right V1, in the left postcentral gyrus (Figure 2, Table 4); with the left V2, in the left superior parietal lobule/BA 7 (Figure 3, Table 4); with the right V2, in the pons and left postcentral gyrus (Figure 4, Table 4); with the left hOC3d, in the right declive and left superior frontal gyrus/BA 8 (Figure 5, Table 4); with the right hOC3d, in the left superior parietal lobule/BA 7 and right supplementary motor area/BA 6 (Figure 6, Table 4); with the left hOC3v, in the left rectus (Figure 7, Table 4); with the right hOC3v, in the left superior frontal gyrus/BA 9 and right superior parietal lobule (Figure 8, Table 4); with the left hOC4d, in the left middle frontal gyrus/BA 9, left supplementary motor area, left superior frontal gyrus/BA 8, and left superior frontal gyrus (Figure 9, Table 4); with the right hOC4d, in the right superior frontal gyrus, left middle frontal gyrus/BA 9, right superior parietal lobule/BA 7, and left superior parietal lobule/BA 7 (Figure 10, Table 4); with the left MT+, in the left superior frontal gyrus/BA 8 (Figure 12, Table 4); with the right MT+, in the left cerebellum posterior lobe (Figure 13, Table 4); with the left FG, in the left middle temporal gyrus (Figure 14, Table 4); and with the right FG, in the right superior frontal gyrus (Figure 15, Table 4). All these figures were edited to save the space and better illustrate the results, and the original figures could be found in the supplemental files.

**TABLE 4:** increased FC after operation.

Seed	Brain region	Peak intensity	Peak MNI coordinate	Cluster size (voxels)
V1 (L)	Superior Parietal Lobule /BA 7 (L)	6.0865	−21 −66 51	58
	Superior frontal gyrus/BA 8 (R)	9.351	3 30 57	127
V1 (R)	Postcentral gyrus (L)	7.5336	−21 −36 78	44
V2 (L)	Superior parietal lobule/BA 7 (L)	7.6319	–9 −66 54	80
V2 (R)	Pons	12.7671	–6 −3 −30	43
	Postcentral gyrus (L)	7.4798	–9 −39 69	64
hOC3d (L)	Declive (R)	9.1414	27 −90 −24	60
	Superior frontal gyrus/BA 8(L)	7.6065	–9 36 57	113
hOC3d (R)	Superior parietal lobule/BA 7 (L)	9.9218	–24 −69 51	46
	Supplementary motor area/BA 6 (R)	8.3583	6 −18 78	52
hOC3v (L)	Rectus (L)	6.6371	−9 27 −18	42
hOC3v (R)	Superior frontal gyrus/BA 9 (L)	6.8125	–18 39 39	50
	Superior parietal lobule (R)	7.6376	15 −78 45	126
hOC 4d (L)	Middle frontal gyrus/BA 9 (L)	14.1715	−48 21 39	42
	Supplementary motor area (L)	8.3222	0 24 45	80
	Superior frontal gyrus/BA 8 (L)	7.3606	–9 45 54	85
	Superior frontal gyrus (L)	7.4915	–36 21 54	63
hOC 4d (R)	Superior frontal gyrus (R)	9.3973	6 18 33	61
	Middle Frontal gyrus/BA 9 (L)	6.7811	–27 33 39	51
	Superior parietal lobule/BA 7 (R)	7.2861	21 −66 63	65
	Superior parietal lobule/BA 7 (L)	6.5869	–24 −69 57	44
MT (L)	Superior frontal gyrus/BA 8 (L)	9.4404	–3 39 51	75
MT (R)	Cerebellum posterior lobe (L)	7.4025	–36 −57 −36	42
FG (L)	Middle temporal gyrus (L)	8.2505	–36 −9 −18	79
FG (R)	Superior frontal gyrus (R)	7.7236	15 15 48	50

**FIGURE 11:**
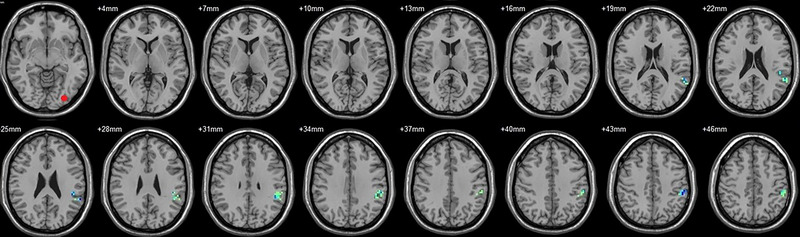
Brain regions showed significantly different FCs with the left hOC4v in STs (postop vs. preop). The red circle in the left upper corner indicates the seed region.

**FIGURE 12:**
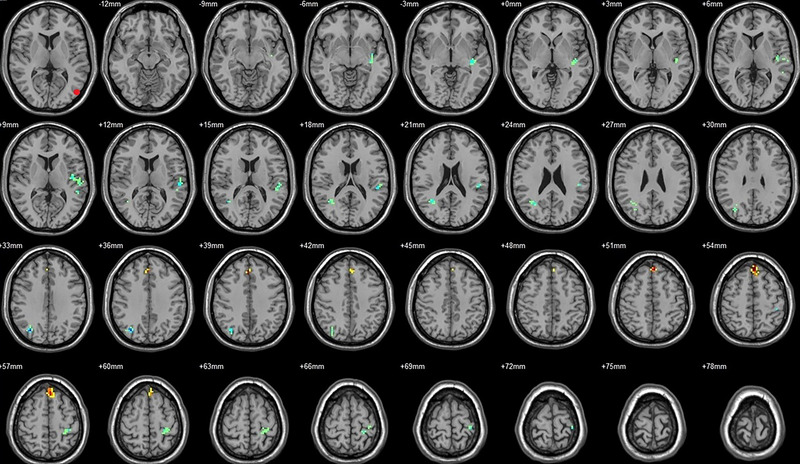
Brain regions showed significantly different FCs with the left MT+ in STs (postop vs. preop). The red circle in the left upper corner indicates the seed region.

**FIGURE 13:**
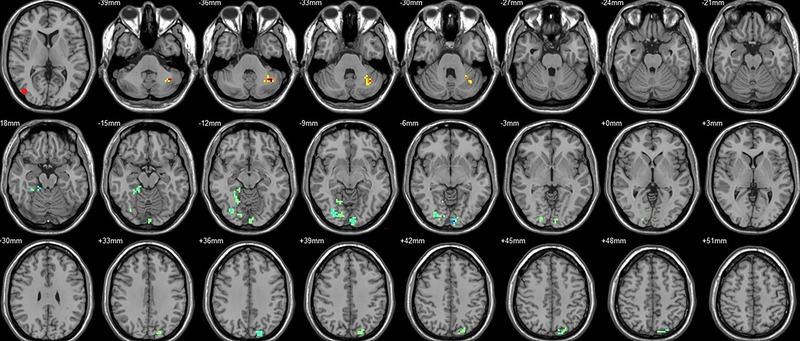
Brain regions showed significantly different FCs with the right MT+ in STs (postop vs. preop). The red circle in the left upper corner indicates the seed region.

**FIGURE 14:**
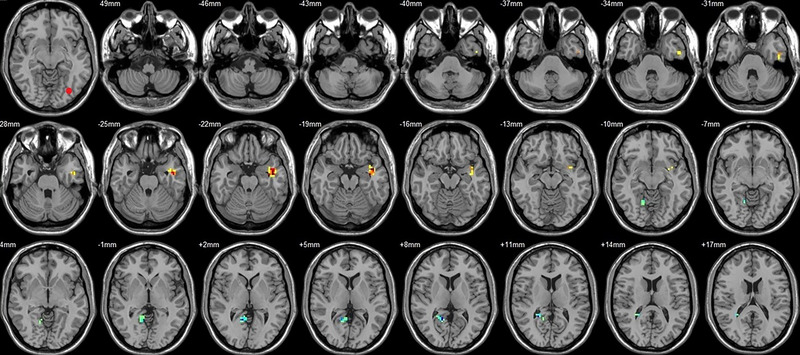
Brain regions showed significantly different FCs with the left FG in STs (postop vs. preop). The red circle in the left upper corner indicates the seed region.

**FIGURE 15:**
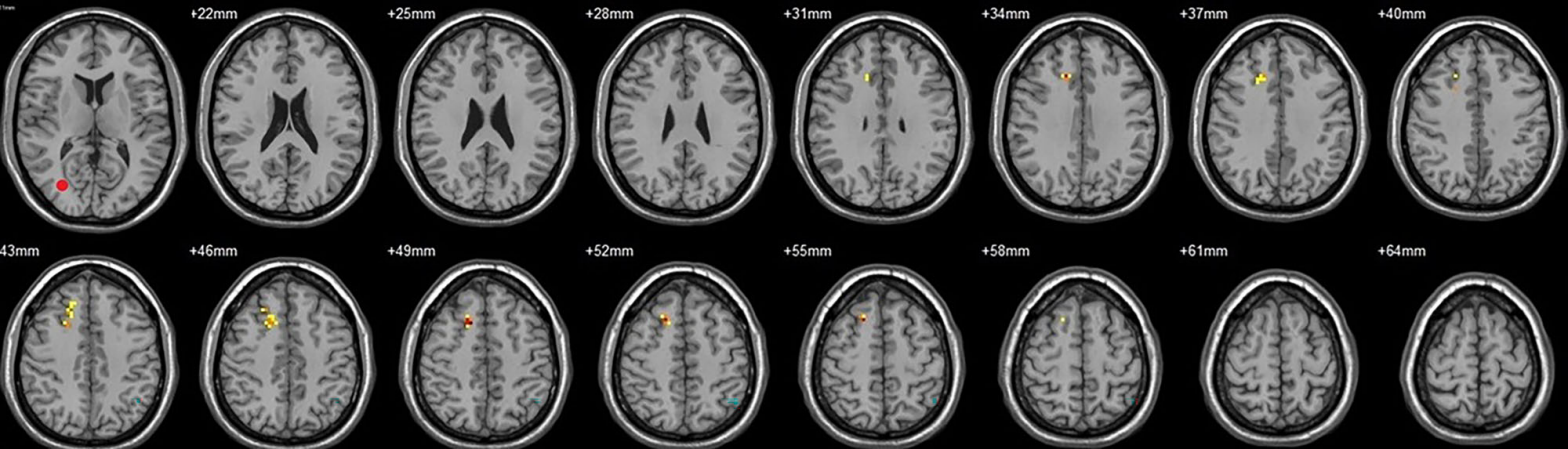
Brain regions showed significantly different FCs with the right FG in STs (postop vs. preop). The red circle in the left upper corner indicates the seed region.

## 4 DISCUSSION

RS‐fMRI is a noninvasive method for evaluating neuronal function during rest in patients. FC is a widely used metric in RS‐fMRI studies, providing high spatial resolution and specificity in detecting changes in neurophysiological signals (Shmuel et al., 2007). In this study, we investigated FC in the visual resting‐state networks of ST patients with AVD after surgery. Our data revealed both increases and decreases in FC among the brain network.

Specifically, we found decreased FC between V1, V2, hOC3d, hOC4d, hOC4v, MT+ and FG in the right middle temporal gyrus, right Brodmann area 19, left Brodmann area 18, left lingual gyrus, left inferior parietal lobule, right middle occipital gyrus, right fusiform, and right lingual gyrus. These results suggest a decreased FC in both the dorsal and ventral visual streams. The dorsal stream is responsible for detecting motion and object location (Merigan & Maunsell, 1993; Tootell et al., 1998). Previous studies have shown abnormal function in the dorsal visual pathway in amblyopia (Backus et al., 2001) and exotropia (Yan et al., 2010). Consistent with these findings, our data show decreased FC between left V1 and right middle temporal gyrus, right hOC3d and left Brodmann area 18, right hOC4d and left lingual, right MT+ and right middle occipital gyrus, and bilateral lingual gyrus, indicating disconnection in the dorsal visual network. The ventral stream, which processes object quality (Kravitz et al., 2013), has also been shown to have decreased FC in patients with damaged vision (Qian et al., 2016) and disrupted visual pathways in amblyopia subjects (Aaen‐Stockdale & Hess, 2008; Simmers et al., 2005). Our data show decreased FC between left FG and right lingual, indicating disconnection in the ventral visual network. These results suggest that AVD may lead to degeneration of the visual pathway and cortex. However, previous studies have shown increased FC within both the dorsal and ventral visual streams in CB (Heine et al., 2015) and the blind brain, indicating anatomical reorganization of nonvisual signals transferred to visual areas (Kupers et al., 2011). Thus, we hypothesize that AVD patients may exhibit a different FC pattern in both the dorsal and ventral visual streams compared to CB.

Although the dorsal and ventral streams were previously thought to function independently, more and more evidence suggests that they are not entirely independent (Schenk & McIntosh, 2010). Studies have shown that there is less interconnection between the two streams in the CB brain than in the normal sighted brain, and this is due to the increased FC within the two streams (Heine et al., 2015). Our data showed decreased FC between left hOC4v and left inferior parietal lobule, as well as right MT+ and right fusiform, which is different from the increased FC found within the two streams in previous studies. One possible explanation for this discrepancy is the different groups recruited for these studies. Another possible explanation is that MT+ may play a compensatory role for the decreased visual signal from the anterior visual pathway, which may lead to the decrease in FC between MT+ and the ventral pathway. Additionally, the processes of cross‐modal nonvisual neural activity in extrastriate cortex may also lead to decreased FC between the two streams (Heine et al., 2015). In a similar study but with different subjects, Qian et al. (2016) also showed decreased FC between the two streams in LPDV patients. Taken together, we speculate that the decreased visual information from the anterior visual pathway may cause less interconnection between the two streams in different types of groups such as the CB, AVD, and LPDV. We will design a study to investigate the interconnection between the two streams in the future.

Compared with the preoperative counterparts, our study observed decreased FC with V1, V2, hOC3d, and MT+ in the right superior temporal gyrus/BA38, right middle temporal gyrus, right Brodmann area 19, left Brodmann area 18, and right middle occipital gyrus. These results may imply decreased FC in the third visual pathway. The third pathway originates from the early visual cortex, via motion‐selective areas, and projects into the superior temporal sulcus (STS). The STS may process the functions of moving faces and bodies, and this pathway is assumed to be involved in social perception (Pitcher & Ungerleider, 2021). In contrast to our study, Qian et al. (2016) showed an increase in FC between MT+ and bilateral superior temporal gyrus in PA patients with damaged vision, and Heine et al. (2015) showed no significant difference in FC between MT+ and superior temporal gyrus in CB. These different results may be explained by the fact that most patients in the first study suffered from damaged vision for a long time, so MT+ may have compensated for the decreased visual signals from V1, leading to the reorganization of visual activity in the regions of the anterior visual pathway. However, in our study, the patients suffered from AVD, and compensation might not have occurred in a timely manner, leading to persistent decreased FC. The second study showed no FC change in the third stream, increased FC within, and decreased FC between the ventral and dorsal streams in the CB. Sliwinska et al. (2020) demonstrated that face recognition is maintained in the third stream, even when face‐selective areas have been damaged in the ventral pathway. All these findings may imply some kind of balance that occurs among the three pathways in the CB. Therefore, we hypothesize that the response in the third visual pathway may differ in different types of visual deprivation due to the underlying mechanisms of vision neuroplasticity. More fMRI studies are needed in the future.

Our data reveal no FC alteration between V1 and V2 in AVD patients with ST. Heine et al. (2015) also found no changes in FC between V1 and V2 in CB, and similar results were presented in EB (Butt et al., 2013; Burton et al., 2014). Qin et al. (2015) suggested that early visual deprivation could cause more extensive brain functional reorganization than late deprivation, implying enhanced nonvisual information processing function in CB. However, in our study, as our subjects belong to late AVD, we hypothesize that AVD might cause stronger top‐down modulation (Murphy et al., 2016), enhancing FC within the primary vision region. An alternative explanation may be related to the special subjects in our study. Most of the patients suffered impaired vision before surgery, and the FC between V1 and V2 might have decreased preoperatively (Qian et al., 2016). After surgery, the acute decreased vision input might cause decreased FC, but the decreased FC is not lower than presurgery significantly, so no FC change can be observed between the V1 and V2. As the literature on changes in FC of primary visual areas in vision‐deprived individuals is incongruent, future well‐designed studies should be performed.

In our study, decreased FC with right hOC3v and bilateral MT+ was identified in the right Brodmann area 41, postcentral gyrus/BA 3, and precuneus/BA 7. These areas are parts of a broader action observation network (AON) (Caspers et al., 2010). Watching others’ activities leads to a reaction in the AON (Cross et al., 2009; Gazzola et al., 2007), and it is assumed to evaluate others’ activities (Rizzolatti & Craighero, 2004). The AON is related to social and motor skills (Fishman et al., 2015; Williams et al., 2017). Our results show lessened FC between the visual region and the substrate of AON after AVD, and the AON may be involved in neural reorganization for vision deprivation.

Our data show that decreased FC with MT+ involves more regions than other ROIs. MT+ is the key region of the dorsal pathway and the third pathway mentioned above. The thalamus‐MT bypass may play a compensatory role when vision information decreased from the anterior vision pathway (Mascioli et al., 2012). MT+ may be a potential substitute when a lesion in V1 occurs at an early age (Bridge et al., 2008; Werth, 2006). MT was reported to respond to auditory and tactile motion in CB (Ricciardi et al., 2007). MT may be specialized in the procession of motion vision (de Jong et al., 1994; Tootell et al., 1995). Therefore, blindness might cause a multimodal response in the MT region. Our results may imply that coexisting neural connections between MT+ and relative areas were mostly affected by AVD. MT+ may play an important role in neural plasticity in case of AVD.

Decreased FC with V1 and V2 was observed in the midbrain and cerebellum (declive), while increased FC with right V2, left hOC3d, and right MT+ was observed in the pons, right declive, and left cerebellum posterior lobe. However, Heine et al. (2015) showed that only stronger connectivity patterns between hOC3d and hOC4v and the cerebellum were found in CB (). Moreover, Qian et al. (2016) revealed no change in FC between the visual region and the cerebellum in late vision‐deprived patients. The cerebellum has an interaction with the frontal eye fields (Kelly & Strick, 2003; Middleton & Strick, 2001), and damage to the cerebellum can cause abnormal eye movement (Straube et al., 1997). Therefore, we speculate that AVD leads to decreased cerebellar function, causing reciprocal increased connections of the cerebellum due to compensation and reorganization of neural activity. Subsequently, the FC of the cerebellum enhances and returns to baseline levels in the late stage. More studies should be performed in the future.

“Top‐down” refers to cognitive influences and higher‐order representations that impinge upon earlier steps in information processing. The top‐down signal can integrate the function between objects and backgrounds in the visual field to maintain a stable representation of the objects within it (Gilbert & Li, 2013). Our results show that increased FC with V1, V2, hOC3d, hOC3v, hOC4d, MT+, and FG was observed in the bilateral superior frontal gyrus/BA8, bilateral superior parietal lobule/BA7, bilateral supplementary motor area/BA6, left postcentral gyrus (L), left middle temporal gyrus, and left middle frontal gyrus/BA9. All these areas might be involved in top‐down modulation as feedback by decreased FC within and between visual pathways after AVD. Qian et al. (2016) also observed “top‐down” influences in LPDV patients. However, bilateral superior parietal lobule/BA7 and supplementary motor area/BA6 were not involved in top‐down modulation in their results when compared with ours. Heine et al. (2015) revealed the “top‐down” influences in CB. However, superior frontal gyrus/BA8 and supplementary motor area/BA6 were not in top‐down modulation when compared with ours. Therefore, it seems that the supplementary motor area/BA6 is the only region related to “top‐down” in our study. Based on this analysis, we speculate that “top‐down” influences play an important role in the reorganization of neural function in vision deprivation, and the high‐order regions involved in “top‐down” may differ in different types of vision deprivation. The supplementary motor area/BA6 may be a unique node in AVD compared to other types of vision damage. Our data show that increased FC with 7 ROIs was observed in the superior frontal gyrus/BA8, 5 ROIs in the superior parietal lobule/BA7, and 2 ROIs in both the supplementary motor area/BA6 and middle frontal gyrus/BA9. All these may suggest that the superior frontal gyrus/BA8 exerts the most projection, implying that the superior frontal gyrus/BA8 is the most important high‐order region involved in “top‐down” influences in the case of AVD. It would be interesting for future studies to examine the “who” and “how” of the “Top‐down” influences in AVD. Our data show that the increased FC with hOC4d involves more high‐order regions than other ROIs. This suggests that hOC4d receives the most projection among the early‐stage vision regions, indicating that hOC4d may be the key low‐order region influenced by “Top‐down” signals when vision function is affected by AVD. These findings suggest that certain hot zones may arise during “Top‐down” modulation. As we have shown that MT+ is mostly affected by AVD, we hypothesize that the hOC4d‐MT+ complex may act as a hub in the vision network during AVD. However, the mechanism of interactions between the hOC4d‐MT+ complex and other regions after AVD needs to be elucidated further.

Compared to their preoperative counterparts, patients showed increased FC with the left hOC3v in the left rectus. The rectus gyrus is part of the ventromedial prefrontal cortex (vmPFC), which is involved in social and cognitive activities (Hiser & Koenigs, 2018). Our results suggest that the increased response of the rectus gyrus is related to neurofunctional reorganization after AVD.

There are some limitations to this study. First, the small number of patients in this study limits its generalizability. Second, we only assessed visual acuity for ophthalmic evaluations as it was easy to conduct in a clinical setting, but it was difficult to quantify the visual field. Future studies will evaluate visual impairment more comprehensively. Thirdly, healthy control subjects were not recruited, which could limit the interpretation of the results. Fourthly, the lateralization of the visual defect should be taken into consideration and functional connectivity analyses should separate ipsilateral and contralateral effects. But in our study, the number of the patients is small, it is hard to make such analysis. So, we will collect more cases to make further analyses in future. Lastly, we only used RS‐fMRI to explore FC because it is convenient to perform in a clinical setting. Future studies will perform multimodal neuroimaging analysis to investigate the interaction within brain regions further.

## 5 CONCLUSIONS

To summarize, our study demonstrates that patients with AVD after surgery experience neurofunctional reorganization in the vision‐related cortex. We observed decreased FC in most subareas of the dorsal, ventral, and third visual pathways, suggesting degeneration of the visual pathway. However, we also observed compensatory mechanisms in the form of decreased FC in MT+, increased FC in some higher‐order cortex regions, particularly the superior frontal gyrus/BA8 and in the vision regions of early stage, especially the hOC4d. These findings suggest that “Top‐down” influences play an important role in the processing of visual information after AVD. Future studies are needed to investigate the neural plasticity mechanisms within the visual cortex and the interaction between visual and higher‐order cortex in patients with specific visual impairments.

## AUTHOR CONTRIBUTIONS

FW and TZ contributed equally to this work. FW drafted the paper. FW and JJ designed the study. FW analyzed the data and interpreted the experiments. JJ performed critical revision. PW and YZ conducted the data analysis. JJ performed part of the experiments. All authors read and approved the final manuscript.

## CONFLICT OF INTEREST STATEMENT

The authors declare that the research was conducted in the absence of any commercial or financial relationships that could be construed as a potential conflict of interest.

### PEER REVIEW

The peer review history for this article is available at https://publons.com/publon/10.1002/brb3.3462.

## Data Availability

The data that support the findings of this study are available from the corresponding author upon reasonable request.
